# Alterations of Plasma Galectin-3 and C3 Levels in Patients with Parkinson’s Disease

**DOI:** 10.3390/brainsci11111515

**Published:** 2021-11-15

**Authors:** Hsiu-Chuan Wu, Kuo-Hsuan Chang, Mu-Chun Chiang, Chiung-Mei Chen

**Affiliations:** 1 Department of Neurology, Chang Gung Memorial Hospital, College of Medicine, Chang Gung University, 5 Fuhsing St., Gueishan, Taoyuan 333, Taiwan; serenawu@cgmh.org.tw (H.-C.W.); gophy5128@cgmh.org.tw (K.-H.C.); 2 General Practice, Long Lane Medical Center, St Helens and Knowsley Teaching Hospitals NHS Trust, Warrington Rd., Rainhill, Prescot L35 5DR, UK; muchunchiang@hotmail.com

**Keywords:** Parkinson’s disease, complement, phagocytosis, galectin-3, C3, biomarker

## Abstract

Parkinson’s disease (PD) is characterized by progressive neurodegeneration of dopaminergic neurons in the ventral midbrain. The complement-phagosome pathway is involved in the pathogenesis of PD. Here we measured levels of complement-phagocytosis molecules, including galectin-3, C3, C4, and cathepsin D, in the plasma of 56 patients with PD, and 46 normal controls (NCs). Plasma levels of galectin-3 (9.93 ± 3.94 ng/mL) were significantly higher in PD patients compared with NCs (8.39 ± 1.95 ng/mL, *p* = 0.012), and demonstrated a positive correlation with Hoehn and Yahr stages in PD patients (R^2^ = 0.218, *p* < 0.001). On the other hand, plasma C3 levels were significantly lower in PD patients (305.27 ± 205.16 μg/mL) compared with NCs (444.34 ± 245.54 μg/mL, *p* = 0.002). However, the levels did not correlate with Hoehn and Yahr stages (R^2^ = 0.010, *p* = 0.469). Plasma levels of C4 and cathepsin D in PD patients were similar to those in NCs. Our results show possible altered complement-phagocytosis signals in the peripheral blood of PD patients, highlighting the potential of galectin-3 as a biomarker of PD.

## 1. Introduction

Parkinson’s disease (PD) is a neurodegenerative disease characterized by rigidity of limbs, tremors at rest, slowness of movements, postural instability, and gait disturbance [1]. These clinical presentations are caused by the progressive degeneration of dopaminergic neurons and the presence of eosinophilic cytoplasmic inclusion bodies (Lewy bodies) in the substantia nigra of the ventral midbrain [2]. Current evidence suggests that the misfolded aggregations of α-synuclein, mostly in Lewy bodies, play a central role in the pathogenesis of PD. The misfolded α-synuclein aberrantly activates the complement-phagosome and other pathways, which may contribute to neurodegenerative processes in PD [3]. The complement system is a well-characterized cascade that can be activated by misfolded proteins [4]. The bindings of activated complements to their cell surface receptors in macrophages or microglia elicit responses to the removal of misfolded proteins by phagocytosis [4]. Complement activation is up-regulated in the brains of PD patients. The level of C3 is increased in Lewy bodies and nigral neurons in PD patients [5]. Complements and their receptors, particularly C3 and complement receptor 4 (CR4), could be involved in the clearance of α-synuclein by microglia [6]. The internalized α-synuclein could be transported to phagolysosomes and degraded by cathepsin D, a lysosomal aspartic endo-protease [7]. Entrance of aggregated α-synuclein in microglia also activates the nuclear factor kappa-light-chain-enhancer of activated B cells (NF-κB) [8], which up-regulates galectin-3 [9]. Galectin-3 also activates NF-κB through a positive feedback effect [9]. Suppression of cathepsin D or galectin-3 ameliorates microglia-mediated neurodegeneration [9,10]. Therefore, the neurodegeneration in PD may depend on microglia, exerted through an aberrant activation of the complement-phagosome pathway.

Given the important role of complement-phagosome pathway in the pathogenesis of PD, a few molecules in this pathway have been measured in body fluids. A proteomic study showed C3 and C4 levels were reduced in the cerebrospinal fluids (CSF) of PD patients [11]. Serum galectin-3 levels were significantly elevated in PD patients [12,13]. However, the correlations between these molecules and disease status have not been studied. Here, we characterized the plasma profile of four complement-phagosome molecules, including galectin-3, C3, C4, and cathepsin D, in patients with PD. These levels were also compared with those in normal controls (NCs), as well as correlated with the disease stage.

## 2. Materials and Methods

### 2.1. Ethical Approval

The collecting of clinical information and venous blood from the participants in this study was approved by the Institutional Review Board of Chang Gung Memorial Hospital (ethical license No: 201701921A3, 201801049A3 and 201801051A3).

### 2.2. Patient Recruitment

Patients were recruited from outpatient clinics in neurology, Chang Gung Memorial Hospital-Linkou Medical Center, Taiwan. The recruited patients had a diagnosis of PD which was confirmed by neurologists (H-C Wu, K-H Chang, and C-M Chen) who specialize in movement disorders using the UK PD Society Brain Bank clinical criteria [14]. Hoehn and Yahr (H&Y) stage [15] and levodopa equivalent daily dose (LEDD) [16] were recorded for each patient at the time of recruitment. NCs, frequency matched for sex and age, were recruited via convenient sampling of individuals from outpatient clinics in neurology. The participants did not have any co-morbidities including renal, cardiac or liver dysfunctions, systemic infections, autoimmune diseases, malignancy, and neurological diseases.

### 2.3. Sample Collection

Venipuncture was performed on all participants with blood samples kept at room temperature for 30 min and centrifuged at 1000–2000× *g* for 10 min. Plasma was carefully collected from the supernatant. All collected plasma samples were frozen at −80 °C until analysis

### 2.4. Enzyme-Linked Immunosorbent Assays for Quantification of Inflammatory Marker Expressions in Plasma

We used enzyme-linked immunosorbent assay kits to evaluate the plasma levels of galectin-3 (Human Galectin-3 Immunoassay, R&D Systems, Minneapolis, MN, USA), C3 (Human Complement C3 ELISA Kit, Abcam, Cambridge, UK), C4 (Complement C4 Human ELISA Kit, Abcam, Cambridge, UK), and cathepsin D (Cathepsin D Human ELISA Kit, Abcam, Cambridge, UK). Each assay was measured in duplicate for each sample at the same time.

### 2.5. Statistical Analysis

Prism 8 (GraphPad) was used for statistical analyses. The D’Agostino–Pearson test [17,18] revealed that galectin-3, C3, C4, and cathepsin D were normally distributed. Therefore, the Student’s *t* test was applied to compare the differences in these noncategorical variables among PD and NC groups. A Bonferroni adjustment was applied to correct for multiple comparisons. For the variables with significant differences among PD and NCs, Pearson’s correlation was applied to evaluate the relationship between their levels and H&Y stages. Data are expressed as mean ± standard deviation (SD). All *p-*values were two-tailed and *p* < 0.013 was considered significant.

## 3. Results

**Figure 1:**
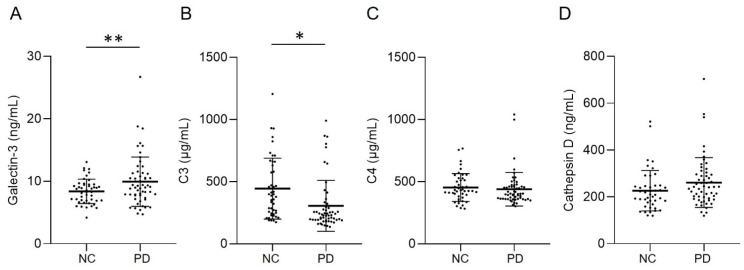
Plasma levels of (**A**) galectin-3, (**B**) C3, (**C**) C4, and (**D**) cathepsin D in patients with Parkinson’s disease (PD, *n* = 56) and normal controls (NCs, *n* = 46). Lines depicted the mean ± standard deviation (SD) of each group. Statistically significant differences between two groups: * *p* = 0.002, ** *p* = 0.012.

**Figure 2:**
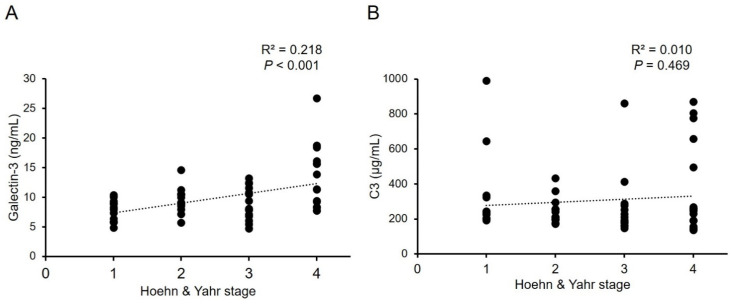
Correlations between serum levels of (**A**) galectin-3, or (**B**) C3, and Hoehn and Yahr stages in patients with Parkinson’s disease. R^2^: Pearson’s correlation coefficient. (*n* = 56).

The demographic data of all groups were displayed in Table 1. H&Y stage and LEDD in PD patients are 2.57 ± 1.13 and 757.40 ± 524.01 mg, respectively. Patients with PD showed higher levels of galectin-3 (9.93 ± 3.94 ng/mL, Figure 1A) compared with NCs (8.39 ± 1.95 ng/mL, *p* = 0.012). Plasma level of C3 in patients with PD (305.27 ± 205.16 μg/mL, Figure 1B) was significantly lower compared with NCs (444.34 ± 245.54 μg/mL, *p* = 0.002). Plasma levels of C4 (PD vs. NC: 439.64 ± 135.04 μg/mL vs. 452.68 ± 112.13 μg/mL, *p* = 0.602) and cathepsin D (PD vs. NC: 261.33 ± 106.16 ng/mL vs. 226.65 ± 86.26 ng/mL, *p* = 0.080) were similar between patients with PD and NCs (Figure 1C,D). With the estimated standard deviations at the level of 0.05, the sample sizes achieved a power of 0.728 and 0.865 for galectin-3 and C3, respectively, to detect differences between compared groups. We further analyzed the correlation between the galectin-3 and C3 plasma levels and H&Y stages at the point of sample collection (Figure 2). The results showed a modest yet significant correlation between plasma levels of galectin-3 and H&Y stages in the patients with PD (R^2^ = 0.218, *p* < 0.001, Figure 2A). On the other hand, plasma levels of C3 (R^2^ = 0.010, *p* = 0.469, Figure 2B) did not demonstrate correlations with H&Y stages. We also analyzed the correlation of LEDD with the four plasma biomarkers and found no significant correlation.

## 4. Discussion

**Figure 3:**
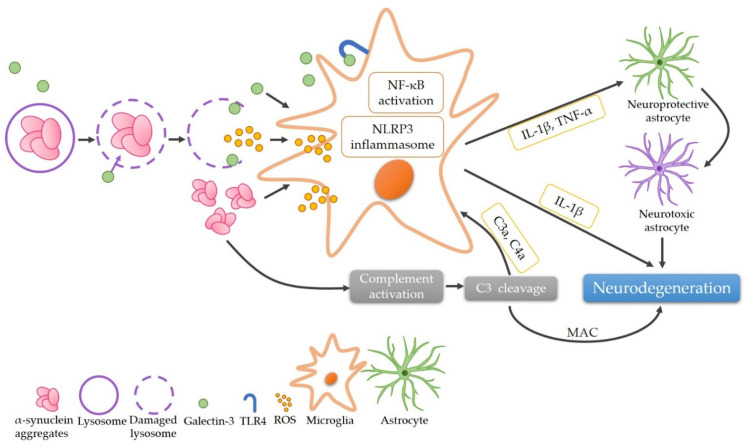
The proposed role of α-synuclein aggregates in the pathogenesis of Parkinson’s disease via the complement-phagocytic pathway. The phagocytosis of α-synuclein leads to the rupture of lysosomes and recruitment of galectin-3, releasing α-synuclein aggregates and ROS that activate microglia subsequently. Galectin-3 further activates microglia via promoting either NF-κB pathway by directly binding TLR4 or the assembly of NLRP3 inflammasomes, resulting in the secretion of IL-1β and TNF-α. These pro-inflammatory cytokines cause neurodegeneration directly or through converting neuroprotective astrocytes to neurotoxic ones. α-Synuclein aggregates also activate the complement system, thereby activating microglia or leading to neurodegeneration through the formation of MAC. (C3: complement component 3; C3a: complement component 3a; C4a: complement component 4a; IL-1β: interleukin 1-beta; MAC: membrane attack complex; NF-κB: nuclear factor kappa-light-chain-enhancer of activated B cells; NLRP3: the NLR family pyrin domain containing 3; ROS: reactive oxygen species; TLR4: Toll-like receptor 4; TNF-α: tumor necrosis factor-alpha).

The complement-phagocytic pathway may play a role in PD pathogenesis. Within this study, we demonstrated that plasma galectin-3 levels are elevated, while C3 levels are reduced in PD patients (Figure 3). Importantly, our data demonstrate a positive correlation between plasma levels of galectin-3 and H&Y stages, strengthening its potential as a biomarker for PD.

The phagocytosis of α-synuclein leads to the rupture of lysosomal membranes and recruiting galectin-3 [19]. Galectin-3 is a multipotent, evolutionarily conserved, cell surface glycoconjugate-crosslinking carbohydrate-binding protein [20]. It is an inflammatory mediator highly expressed in activated microglia [21,22]. Galectin-3 activates the NF-κB pathway, inhibits the clearance of damaged lysosomes and promotes assembly of the NLR family pyrin domain containing three (NLRP3) inflammasomes [9]. Changes of galectin-3 in CSF and blood have been reported in neurodegenerative diseases including Alzheimer’s disease (AD), amyotrophic lateral sclerosis (ALS), and PD [12,13,23]. CSF and serum galectin-3 levels are higher in patients with AD and ALS compared with NCs [23]. PD patients also demonstrate an elevated level of galectin-3 in serum [12,13]. Our findings consistently demonstrate higher galectin-3 plasma levels in PD patients compared to NCs. Importantly, both serum and plasma levels of galectin-3 demonstrates positive correlation with H&Y stages in PD patients [12]. These findings suggest the potential of galectin-3 as a biomarker in monitoring the neurodegenerative progression of PD.

The complement system is well-known as an essential pathway of the innate and adaptive immune systems [24]. In addition to a crucial molecule in both the classical and the alternative complement cascades, C3 is also closely related to the synapse elimination and brain aging [25,26]. Knockout of C3 rescues the region-specific loss of synapses and neurons as well as cognitive decline in aging mice [26]. Evidence also suggests that C3 and its downstream C3d signaling play an important role in PD. The binding of C3d to the Lewy bodies of substantia nigra or brainstem are pronounced in brains of patients with PD and diffuse Lewy body disease [27,28]. Reactive astrocytes identified in PD patients are also immunoreactive to C3 [29]. Moreover, C3d immunoreactivities in the ventral midbrain with robust loss of dopaminergic neurons can be induced by intrastriatally injected α-synuclein fibrils [30]. However, the alterations of C3 in body fluids of PD patients are still controversial. A proteomics study showed that the C3 level was reduced in the CSF of PD patients [31]. In serum, increased levels of C3 were reported in PD patients [32], whereas the other study found lower C3 levels in male PD patients or patients with attention/memory problems [33]. Consistent with our findings, Alberio et al. reported lower plasma levels of C3 in PD patients compared with NCs. The reduction of C3 in PD patients may be caused by the depletion of the activated complement system. However, our results do not demonstrate significant correlation between plasma C3 levels and H&Y stage. Further investigation is necessary to understand the role of C3 in PD pathogenesis.

C4 is a marker of the classical complement pathway [24]. The complement-activated oligodendroglia revealed by anti-C4 antibodies can be observed in the substantia nigra of PD patients [27]. Reduced levels of C4 have been reported in the CSF of PD patients [34]. Alberio et al. also reported low levels of C4 in the plasma of PD patients [35]. However, this was not found in our study. Differences in ethnicity, disease severity or medications may have contributed to this discrepancy. A large cohort will be necessary to clarify the role of C4 as a biomarker for PD. The mutations of *GBA* gene which cause Gaucher disease (GD) are also the most common genetic risk factor of PD. GD is associated with reduced plasma C3 and C4 concentrations [36]. Further study to correlate the *GBA* genotype in PD patient with their plasma C3 or C4 levels will clarify the potential confounding effect.

Cathepsin D is a lysosomal aspartate protease responsible for the degradation of proteins, including α-synuclein [37]. It is widely expressed in the cortex, hippocampus, striatum, and substantia nigra [12]. Cathepsin D knockout mice demonstrate extensive accumulation of α-synuclein in neurons similar to PD patients [38]. Overexpression of cathepsin D is protective against α-synuclein-mediated neurotoxicity in SH-SY5Y neuroblastoma cells, while Y125A mutation at the α-synuclein cleavage site of cathepsin D leads to neuroprotection resistance [38]. In CSF, cathepsin D activities are reduced in PD patients [39]. In this study, we did not find changes of cathepsin D levels in the plasma of PD patients. Although, our study showed a trend toward increased plasma cathepsin D levels in PD patients, there was no statistical significance. This might be due to a smaller sample size in this study. Future studies measuring the cathepsin D activity in peripheral blood is warranted.

There are some limitations in this study. The population selection may be biased as it was a single-center study with a relatively small sample size. The CSF levels of these markers are not available. Further studies clarifying the temporal trend of galectin-3 and C3 in plasma would be important to consolidate their associations with PD. The comparisons of these markers with α-synuclein, and other lysosomal or inflammatory molecules would give more insight in the causality of the pathogenesis of PD.

## 5. Conclusions

Our study demonstrated significantly higher levels of galectin-3 and lower levels of C3 in the plasma of patients with PD compared to the controls. Furthermore, the galectin-3 plasma level correlating positively with H&Y stage could reflect clinical severity. These observations suggest the potential of galectin-3 as a biomarker for PD.

## Figures and Tables

**Table 1:** Demographic characteristics and blood biochemical parameters of patients with Parkinson’s disease (PD) and normal controls (NC).

	NC (*n* = 46)	PD (*n* = 56)
Age (years)	65.91 ± 9.78	64.79 ± 10.15
Age at onset (years)		58.75 ± 10.13
Disease duration (years)		6.04 ± 5.50
Male (%)	25 (54.34)	32 (57.14)
Hoehn and Yahr stage		2.57 ± 1.13
LEDD (mg)		757.40 ± 524.01
Dyskinesia (%)		15 (26.79)
HbA1c (%)	6.51 ± 1.37	6.21 ± 0.68
Creatinine	0.78 ± 0.25	0.81 ± 0.24
AST	26.66 ± 7.75	26.27 ± 12.03
ALT	22.13 ± 10.85	17.51 ± 16.02

ALT: alanine transaminase; AST: aspartate transaminase; HbA1C: glycohemoglobin; LEDD: levodopa equivalent daily dosage; NC: normal controls; PD: Parkinson’s disease.

## Data Availability

All data included in this study are available upon reasonable request by contact with the corresponding author.
